# Potential of M-Wave Elicited by Double Pulse for Muscle Fatigue Evaluation in Intermittent Muscle Activation by Functional Electrical Stimulation for Motor Rehabilitation

**DOI:** 10.1155/2016/6957287

**Published:** 2016-03-27

**Authors:** Naoto Miura, Takashi Watanabe

**Affiliations:** ^1^TESS Co., Ltd., T-Biz 404, 6-6-40 Aramaki-Aza-Aoba, Aoba-ku, Sendai 980-8579, Japan; ^2^Graduate School of Biomedical Engineering, Tohoku University, 6-6-12 Aramaki-Aza-Aoba, Aoba-ku, Sendai 980-8579, Japan

## Abstract

Clinical studies on application of functional electrical stimulation (FES) to motor rehabilitation have been increasing. However, muscle fatigue appears early in the course of repetitive movement production training by FES. Although M-wave variables were suggested to be reliable indices of muscle fatigue in long lasting constant electrical stimulation under the isometric condition, the ability of M-wave needs more studies under intermittent stimulation condition, because the intervals between electrical stimulations help recovery of muscle activation level. In this paper, M-waves elicited by double pulses were examined in muscle fatigue evaluation during repetitive movements considering rehabilitation training with surface electrical stimulation. M-waves were measured under the two conditions of repetitive stimulation: knee extension force production under the isometric condition and the dynamic movement condition by knee joint angle control. Amplitude of M-wave elicited by the 2nd pulse of a double pulse decreased during muscle fatigue in both measurement conditions, while the change in M-waves elicited by single pulses in a stimulation burst was not relevant to muscle fatigue in repeated activation with stimulation interval of 1 s. Fatigue index obtained from M-waves elicited by 2nd pulses was suggested to provide good estimation of muscle fatigue during repetitive movements with FES.

## 1. Introduction

Functional electrical stimulation (FES) has been studied clinically as an application to an orthotic and therapeutic aid in rehabilitation of upper and lower limb motor functions [1–4]. Application of FES to motor rehabilitation has been suggested to reduce the term needed to improve motor functions of paralyzed limbs compared with conventional rehabilitation [2, 4, 5]. Repetitive movement therapy mediated by electrical stimulation has also a possibility to facilitate motor relearning [3, 6]. However, muscle fatigue occurs early in the course of FES training due to muscle activation based on the inverse size-order recruitment, poor fatigue resistance of paralyzed muscles, or high stimulation frequency near the upper values of physiological neural firing rates. The occurrence of early muscle fatigue becomes one of the problems of using FES in motor rehabilitation.

Muscle compound action potential elicited by electrical stimulation (M-wave), which can be measured with surface electrodes, has the potential to be an indicator of muscle fatigue [7]. Peak-to-peak amplitude of M-wave has been found to be a reliable muscle fatigue indicator that characterizes FES-produced force in continuous constant stimulation [8–11]. However, long lasting continuous stimulation used in those previous studies is not common in rehabilitation training. For example, in a gait rehabilitation exercise for motor paralysis, paralyzed leg muscles are activated repeatedly with time interval in accordance with the preprogrammed muscle activation pattern that mimics normal gait [2, 6, 12]. In such rehabilitation training, electrical stimulation pulses are applied to muscles intermittently. In this stimulation condition, the time interval between movement productions by electrical stimulation is considered to rest the electrically stimulated muscle and increases activity of the muscle, which leads to changes in M-waves after the interval between activations [7]. Thus, M-waves have to be tested in fatigue evaluation under the intermittent stimulation condition. The ability of M-wave for muscle fatigue evaluation needs more studies to determine the reliability under the dynamic movement condition. Decrease of muscle torque during fatiguing FES cycling under the isokinetic low-cadence condition could not be predicted adequately by M-wave [13]. On the other hand, in rapid speed FES cycling, peak-to-peak amplitude of the M-wave decreased as cycling speed decreased [14]. These studies in FES cycling showed different property of M-wave in FES-induced muscle fatigue under the dynamic exercise condition.

In this study, M-waves elicited by double pulses were examined in evaluation of muscle fatigue for motor rehabilitation with FES. M-waves elicited by the additional pulse that constituted a double pulse in a stimulus pulse train for FES control were suggested to provide useful information of early muscle fatigue in a continuous, constant electrical stimulation under the isometric condition [11]. Therefore, it is necessary to determine whether M-waves elicited by double pulses are sensitive to detect muscle fatigue induced by FES during intermittent repetitive movement training.

The purpose of this study was to determine the effectiveness of M-waves elicited by double pulses in estimating muscle fatigue during applying intermittent repetitive burst stimulation pulses under both the isometric and dynamic movement conditions considering rehabilitation training. In this paper, first, M-waves elicited by single pulses and by the 2nd pulses of double pulses in stimulation bursts were measured under two intermittent stimulation conditions: force production under the isometric condition and the maximum knee extension angle control by FES. Those M-wave amplitudes were compared in order to find a possibility of M-waves for muscle fatigue evaluation during intermittent stimulation. Then, a muscle fatigue indicator based on M-waves was discussed on muscle fatigue evaluation during repeated dynamic movement training with FES.

## 2. Experimental Methods

### 2.1. Measurements of M-Waves

**Figure 1:**
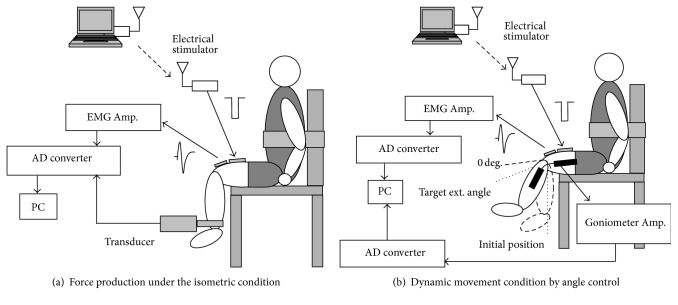
Experimental setup for 2 measurement conditions.

In order to examine M-waves in estimating muscle fatigue during intermittent repetitive stimulation under both the isometric and dynamic movement conditions, M-waves elicited by bursts of electrical stimulation pulses were measured together with produced force or knee joint angle under the two repetitive stimulation conditions: (a) knee extension force production under the isometric condition and (b) dynamic movement condition by knee joint angle control.  Figure 1 shows experimental setup for the 2 measurement conditions. Those measurements were performed with four neurologically intact subjects in the sitting position. The subjects seated in the chair (Musculator GT-30, OG Giken) and relaxed their legs during measurements. Subject's consents to participate in the experiment were obtained.

M-waves were amplified with the EMG amplifier (gain: 46 dB) and low pass filtered with cutoff frequency of 12 kHz in order to reduce influence of stimulation artifact. The knee extension force produced by isometric muscle contraction was measured with a transducer (DTG-20, DIGITECH Co., Ltd.) at the shank above the ankle joint (at about 38 cm from the knee joint axis) under measurement condition (a). Knee joint angle was measured with an electrical goniometer (M180, Penny & Giles) under condition (b). In condition (a), EMG signals and force data were recorded by personal computer through AD converter (USB-6211, National Instruments Corporation) (sampling frequency of 20 kHz). In condition (b), EMG signals were measured by the same way as condition (a) and knee joint angle data were recorded by personal computer through AD converter in wireless module (WCU-241, K2-denshi Inc.) with sampling frequency of 40 Hz by using previously developed measurement system [15].

**Figure 2:**
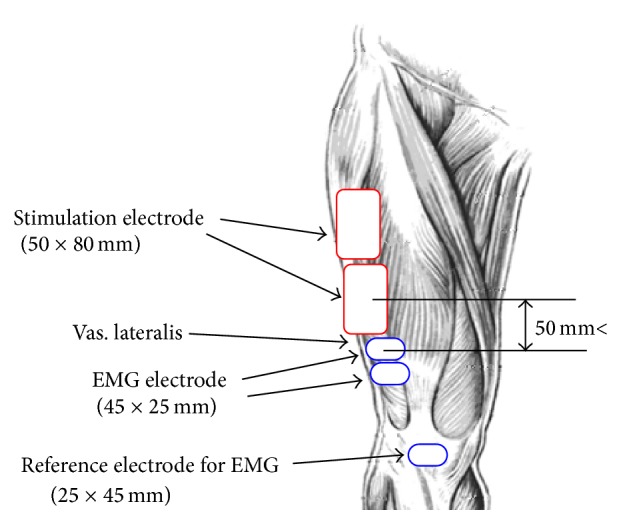
Electrode placement for surface electrical stimulation and M-wave measurement to the vastus lateralis muscle.

Electrical stimulation was applied to the vastus lateralis muscle through surface electrodes (SRH5080, 50 × 80 mm, Sekisui Plastics), in which monophasic pulses with 50 ms period and 0.3 ms width were used. Pulse amplitude was determined to develop full knee extension by a stimulation burst. Electrode placement for surface electrical stimulation and M-wave measurement is shown in Figure 2. The surface stimulation electrodes were placed lengthwise over the middle of the muscle belly with an interelectrode distance (center-to-center) of 85 mm. M-waves were recorded through surface electrodes (F-150M, 25 × 45 mm, NIHON KOHDEN) positioned with an interelectrode distance of 30 mm away from the stimulating electrodes with a distance of more than 50 mm. The reference electrode was placed on the patella of the knee.

**Figure 3:**
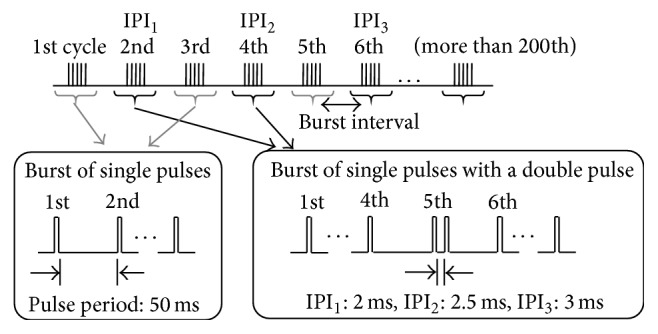
Electrical stimulation pulses applied in order to measure M-waves.

Stimulation burst pulses with and without an additional pulse for a double pulse were applied alternately in one measurement session, in which interpulse interval (IPI) of the double pulse was set to 2 ms, 2.5 ms, or 3 ms and was varied in a measurement trial in order (Figure 3). The additional pulse was inserted into a stimulation burst after the fifth pulse because the shape of M-wave varied in the first few pulses in a burst [16]. Therefore, the 5th pulse and the additional pulse constituted a double pulse.

The 2 measurement conditions are described as follows.

*(a) Knee Extension Force Production under the Isometric Condition.* M-waves and knee extension forces produced by bursts of electrical stimulation were measured under the isometric condition (Figure 1(a)). Burst duration of stimulation pulses was set at 750 ms (15 pulses in a burst), since the burst duration to produce the maximum knee extension angle from the neutral position was more than 500 ms in our preliminary test [16]. The total number of repetitive stimulation cycles was more than 200. Time interval between stimulation bursts was set at 1 s.

**Figure 4:**
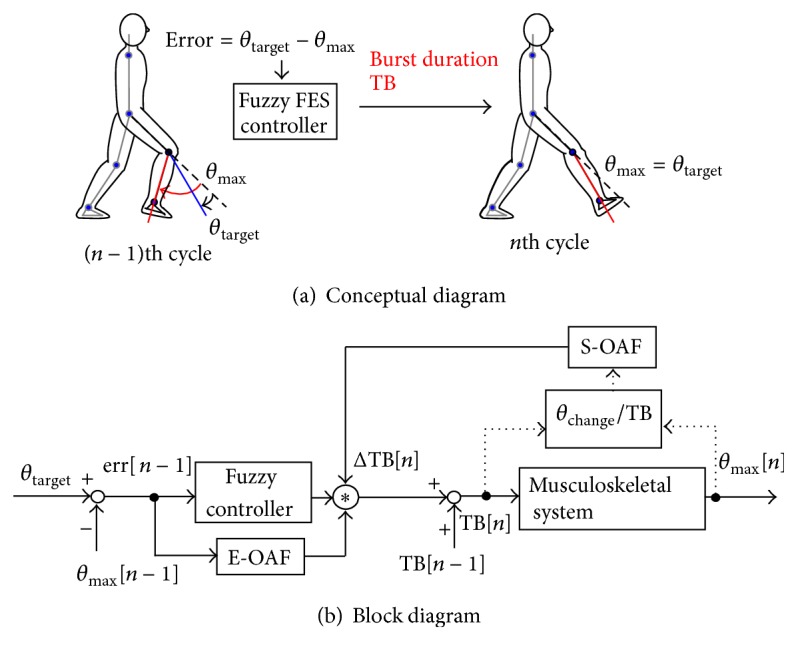
Conceptual diagram of cycle-to-cycle control in controlling maximum knee extension angle in the swing phase of walking (a) and block diagram of the FES control used in this study (b). The maximum knee extension angle was controlled at the sitting position by electrical stimulation based on cycle-to-cycle control in this study. *θ*_target_, *θ*_max_, and *θ*_change_ show the target angle, controlled maximum angle, and joint angle change to single stimulation burst. TB is the burst duration. *θ*_change_/TB shows muscle sensitivity. E-OAF (error-based output adjustment factor) and S-OAF (sensitivity-based output adjustment factor) adjust output of the fuzzy controller in order to improve control ability.

*(b) Dynamic Movement Condition by Knee Joint Angle Control.* M-waves and knee joint angle produced by bursts of electrical stimulation were measured during knee joint angle control (Figure 1(b)). The knee joint angle was controlled by regulating burst duration of stimulation pulses by fuzzy FES controller based on cycle-to-cycle control [15]. The cycle-to-cycle control is a control method of restoring cyclic movements such as gait by using FES as shown in Figure 4(a). Therefore, the cycle-to-cycle control is useful for controlling repetitive movements in FES rehabilitation. In this FES control, burst duration of electrical stimulation of each cycle was determined based on the difference between produced joint angle and the target joint angle. The block diagram of the FES control based on the cycle-to-cycle control used in the experiments is shown in Figure 4(b). Target angle in the control *θ*_target_ was the maximum knee extension angle. The controlled maximum knee extension angle of the previous control cycle *θ*_max_[*n* − 1] is delivered as feedback signal. The burst duration of stimulation pulses of a current control cycle TB[*n*] is regulated basically according to the error in the previous control cycle err[*n* − 1], while pulse amplitude, pulse width, and frequency were fixed.

In measurement of M-waves, the target angle was set at 30 deg. (0 deg. means full knee extension angle). Knee joint angle was measured with electrical goniometer. Control of the 1st control cycle was started when no movement of the knee joint was detected (knee joint angle change became less than or equal to 0.3 deg. for 0.5 s). From the 2nd control cycle, electrical stimulation was applied 0.6 s after the end of the previous stimulation burst, which was determined experimentally from preliminary tests, in order to produce time interval of about 1 s between stimulation bursts.

### 2.2. Data Analysis

**Figure 5:**
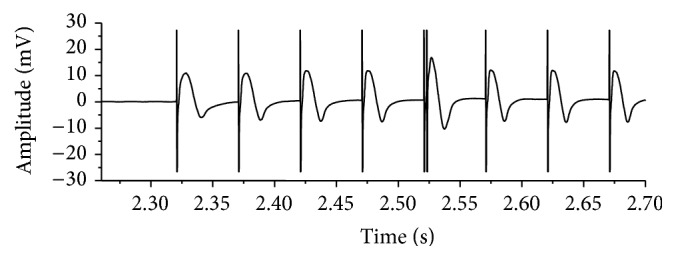
An example of recorded EMG signals in the knee extension force production under the isometric condition (the 2nd stimulation cycle, subject A). The 5th M-wave was elicited by a double pulse with IPI of 2 ms.

**Figure 6:**
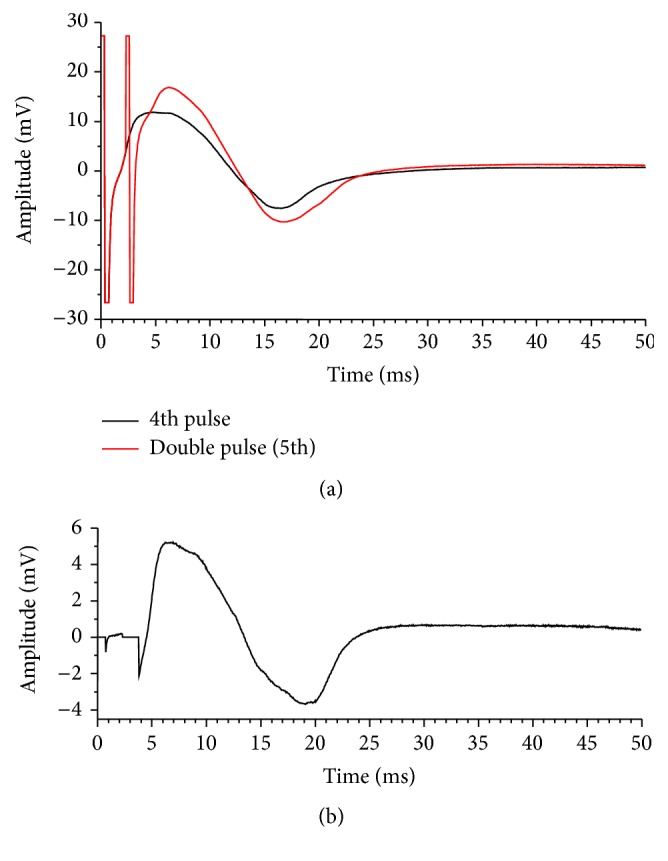
Examples of measured M-waves elicited by the 4th and the double pulse (a) and the calculated M-wave elicited by the 2nd pulse of the double pulse (b) in the same burst duration. The beginnings of M-waves were made to match by the beginning time of the stimulation pulse. Stimulation artifacts in the recorded EMG signals are seen in (a) and those of the 2nd stimulation pulse have been removed in (b).

An example of recorded EMG signal in a stimulation burst is shown in Figure 5 (the first 8 pulses in a stimulation burst). Although stimulation artifact was observed in the measured EMG signal, peak amplitude of M-waves can be detected. The 5th M-wave in Figure 5 was elicited by the double pulse with IPI of 2 ms. M-wave elicited by the 2nd pulse of a double pulse was obtained by subtracting the M-wave elicited by the 4th pulse from the M-wave elicited by the double pulse in the same stimulation cycle. Examples of M-waves elicited by the 4th pulse and the double pulse in the same stimulation burst and the calculated M-wave elicited by the 2nd pulse of the double pulse are shown in Figure 6. The beginning of the M-wave elicited by the double pulse was almost the same as the M-wave elicited by the 4th pulse as seen in Figure 6(a).  Figure 6(b) shows that, in the calculation of M-wave elicited by the 2nd pulse of the double pulse, stimulation artifact caused by the 2nd pulse was removed from the calculated M-wave by setting the M-wave amplitude to 0 V for 1.5 ms from the beginning of the stimulation pulse. Peak-to-peak amplitudes of the M-wave elicited by the 2nd pulse and the M-wave by the 4th pulse in each burst stimulation were calculated.

M-waves were examined in muscle fatigue evaluation comparing with the conventional fatigue index defined by force drop (FI_*f*_(*n*)) [17]. Here, fatigue index calculated from the muscle sensitivity (FI_*S*_(*n*)) was also defined as a reference index, in which the muscle sensitivity was defined as the ratio of joint angle change to stimulation burst duration. Fatigue indexes based on peak-to-peak amplitude of the M-wave by the 4th pulse in a burst (FI_4_(*n*)) and that of the M-wave by the additional pulse of each IPI (FI_ad_^*i*^(*n*)) were defined by following equations:

(1) FIfn=1−FnFmax,FISn=1−SnSmax,FI4n=1−V4nV4max,FIadin=1−VadinVad_maxi,

where *n*, *F*(*n*), and *F*_max_ show the stimulation cycle number, the peak force in the *n*th stimulation cycle, and the maximum value of the produced force in the measurement cycles, respectively. *S*(*n*) and *S*_max_ are the value of the sensitivity at the *n*th stimulation cycle and its maximum value in the measurement. *V*_4_(*n*) and *V*_4max_ are the peak-to-peak amplitude of the M-wave elicited by the 4th pulse in a burst at the *n*th stimulation cycle and its maximum value. *i* means the IPI, that is, the interval for the additional pulse (2 ms, 2.5 ms, or 3 ms). *V*_ad_^*i*^(*n*) and *V*_ad_max_^*i*^ are the peak-to-peak amplitude of the M-wave elicited by the additional pulse with IPI of interval *i* at the *n*th stimulation cycle and its maximum value. In addition, the fatigue index that combined FI_ad_^2^(*n*), FI_ad_^2.5^(*n*), and FI_ad_^3^(*n*) obtained from the M-waves by the additional pulses was defined as

(2) $$\begin{array}{c} FI_{ad}\left(\;m\;\right)=\frac{FI_{ad}^{2}\left(m-2\right)+FI_{ad}^{2.5}\left(m\right)+FI_{ad}^{3}\left(m+2\right)}{3}, \end{array}$$

where *m* is the stimulation cycle number that includes a double pulse with IPI of 2.5 ms.

Pearson correlation coefficient was calculated between fatigue index based on M-wave and conventional fatigue index in order to determine reliability of M-wave for muscle fatigue evaluation.

## 3. Results of Measurement of M-Waves

### 3.1. Knee Extension Force Production under the Isometric Condition

**Figure 7:**
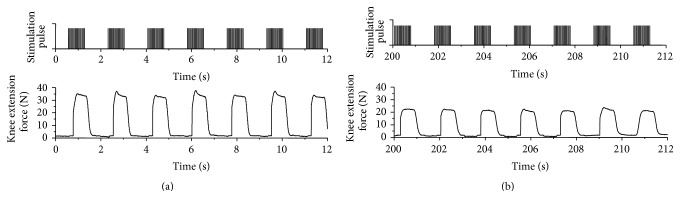
An example of measured knee extension force under the isometric condition. (a) and (b) show produced forces from the 1st stimulation burst and from the 115th burst, respectively.

An example of measured knee extension force is shown in Figure 7 with stimulation pulses. It is found that muscle force produced by electrical stimulation burst decreased as the cycle number increased. In order to evaluate muscle fatigue, maximum force in each produced force by a stimulation burst was detected. Here, since produced force by stimulation burst including double pulse increased peak force values (the 2nd, 4th, and 6th bursts in (a)) as seen in Figure 7, forces produced by single pulse bursts were used in evaluation.

**Figure 8:**
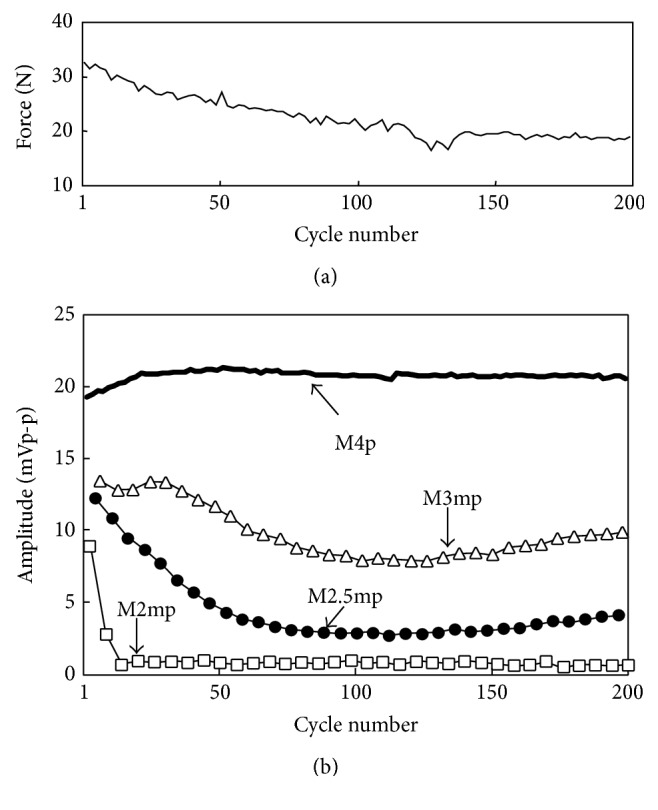
An example of measured M-wave amplitudes during repetitive knee extension force production under the isometric condition (subject A). Force (a) and peak-to-peak amplitude of each M-wave (b) are shown. M4p shows the peak-to-peak amplitude of M-wave elicited by the 4th pulse in a pulse burst, and M3mp, M2.5mp, and M2mp mean amplitudes obtained from the additional pulses with 3 ms IPI, 2.5 ms IPI, and 2 ms IPI, respectively.

Figure 8 shows an example of peak-to-peak amplitude of M-wave and the maximum force under the measurement condition (a). The force decreased as the number of cycles increased because of muscle fatigue caused by the repetitive stimulation. Peak-to-peak amplitude of the M-wave elicited by the 2nd pulse of a double pulse decreased significantly in early cycles, which are shown in Figure 8 by M3mp for 3 ms IPI, M2.5mp for 2.5 ms IPI, and M2mp for 2 ms IPI. In contrast, peak-to-peak amplitude of M-wave elicited by the 4th single pulse in a burst of electrical stimulation pulse (M4p), which was similar M-wave parameter used in previous studies, showed increase in early cycles and slight decrease after that.

### 3.2. Dynamic Movement Condition by Knee Joint Angle Control

**Figure 9:**
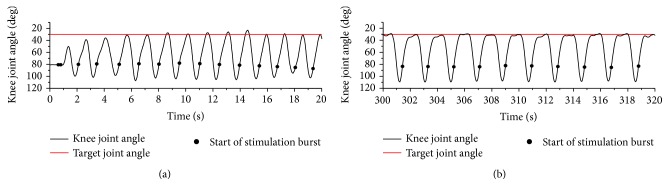
An example of knee joint angle during cycle-to-cycle control (subject A). (a) and (b) plots were results from the 1st control cycle and from the 191st control cycle, respectively. At the 1st control cycle, stimulation burst time was 0 s. Target angle is shown by red line (30 deg.).

Figure 9 shows an example of knee joint angle during cycle-to-cycle control. Knee joint angle at the resting position was about 80 degrees, and the target angle was 30 degrees (0 degrees means full knee extension). Since electrical stimulation bursts were applied 0.6 s after the end of the previous stimulation burst, oscillating movement caused by passive movement after the end of stimulation was not observed. It is found that joint movement changed as the cycle number increased because of muscle fatigue.

**Figure 10:**
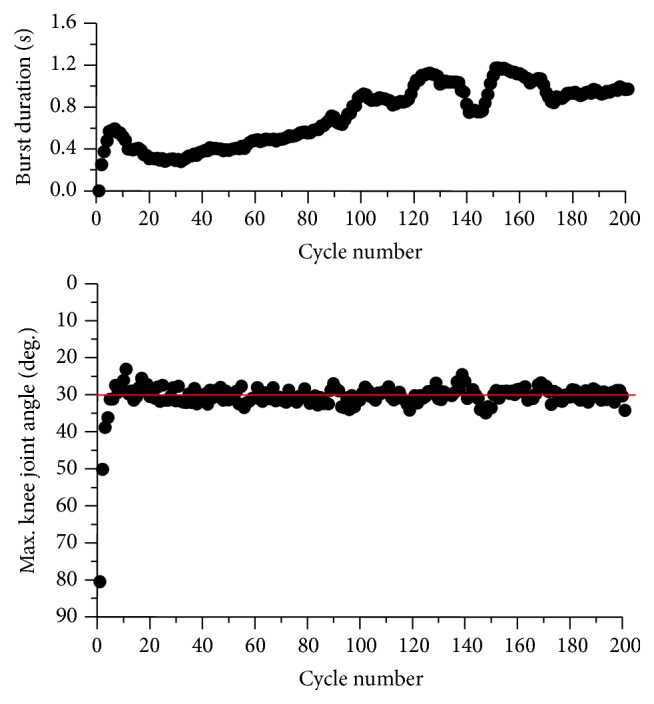
An example of results of cycle-to-cycle control (subject A). Target angle is shown by red line (30 deg.).

An example of control result is shown in Figure 10. Joint angle reached the target angle within the first 5th cycles. The mean error between the maximum knee extension angle and the target angle after reaching the target was 1.5 ± 1.1 deg. These control results were similar to results in our previous report [16], which shows similar movements were achieved in every control cycles. Average values of the number of pulses in a stimulation burst and the time interval between stimulation bursts were 9.8 ± 3.0 pulses and 0.95 ± 0.11 s in average of results with 4 subjects, respectively.

**Figure 11:**
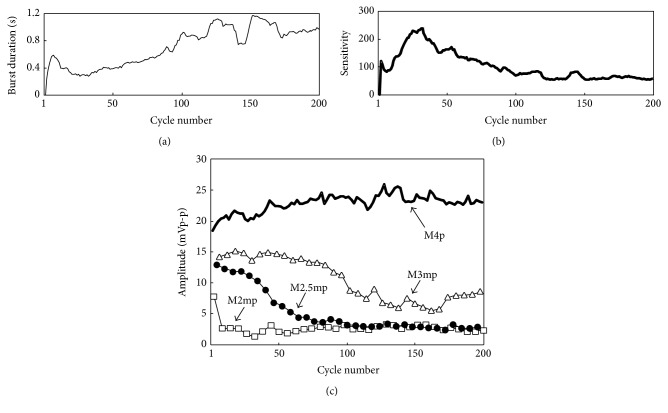
An example of measured M-wave amplitudes during knee extension angle control by fuzzy controller based on the cycle-to-cycle control (subject A). Burst duration (a), sensitivity (b), and peak-to-peak amplitude of each M-wave (c) are shown.

Figure 11 shows an example of the measurement results under the measurement condition (b). The muscle sensitivity increased until about the 30th cycle and afterward decreased. The increase of the sensitivity was considered to be caused by muscle force potentiation [18], and the decrease was caused by muscle fatigue. M3mp, M2.5mp, and M2mp decreased in the early cycles as seen in the measurement condition (a). However, M4p increased gradually as the number of cycles increased.

## 4. Muscle Fatigue Evaluation Using M-Waves

**Figure 12:**
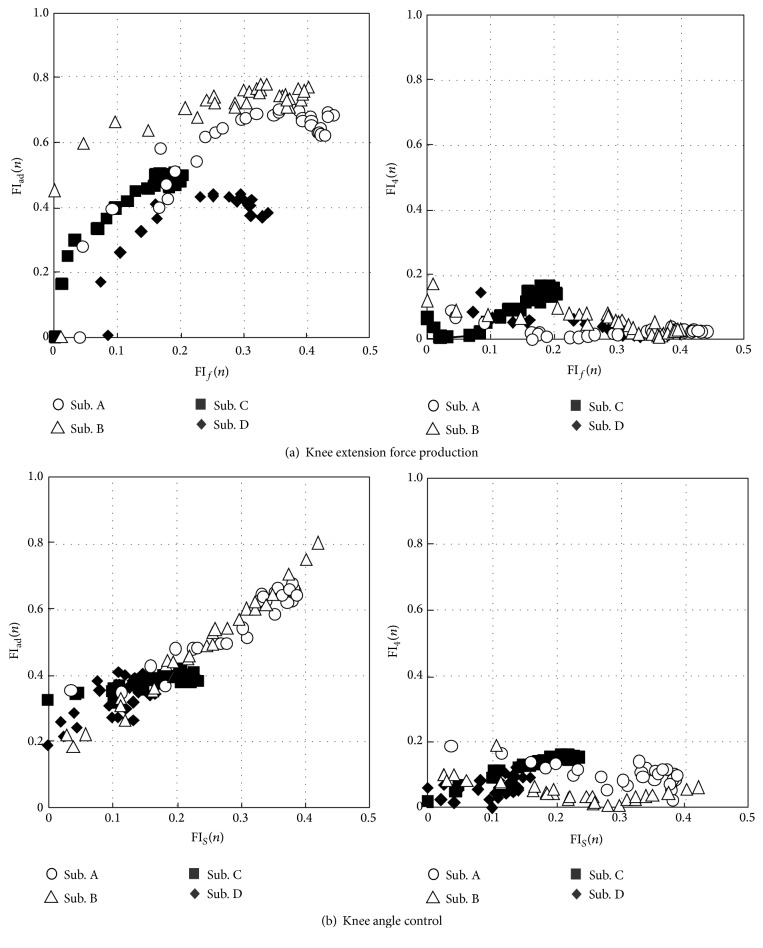
The relationships between fatigue indexes of M-waves (FI_ad_(*m*) and FI_4_(*n*)) and the previous type of fatigue indexes (FI_*f*_(*n*) and FI_*S*_(*n*)).

Figure 12 shows calculated values of fatigue indexes using M-waves (FI_ad_(*m*) and FI_4_(*n*)) in comparison with conventional fatigue indexes using muscle force (FI_*f*_(*n*)) or muscle sensitivity (FI_*S*_(*n*)). For joint angle control, FI_*S*_(*n*) was calculated after the 30th cycle because of increase in the sensitivity in the early cycles. FI_ad_(*m*) increased as FI_*f*_(*n*) or FI_*S*_(*n*) increased. High correlation coefficients were obtained between FI_*f*_(*n*) and FI_ad_(*m*), and between FI_*S*_(*n*) and FI_ad_(*m*), which were 0.74 and 0.94, respectively. In contrast, FI_4_(*n*) was hardly changed with increasing of FI_*f*_(*n*) or FI_*S*_(*n*). Correlation coefficients between FI_4_(*n*) and FI_*f*_(*n*) and between FI_4_(*n*) and FI_*S*_(*n*) were very low (−0.48 and 0.09, resp.).

Since strong correlation was found between FI_*S*_(*n*) and FI_ad_(*m*) as shown in Figure 12(b), a regression equation was calculated by the method of linear least squares in order to estimate fatigue index during the repetitive movement by using the M-waves of the additional pulses (FI_*M*_(*m*)). That is,

(3) $$\begin{array}{c} FI_{M}\left(\;m\;\right)=1.4973\times {FI}_{ad}^{}\left(\;m\;\right)-0.2569. \end{array}$$

**Figure 13:**
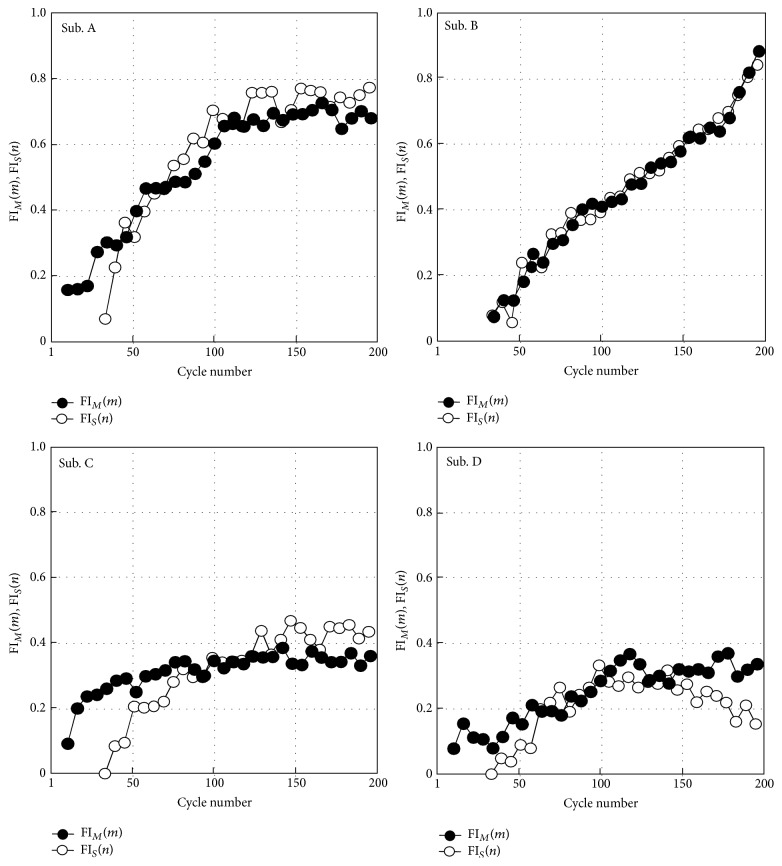
Estimation results of muscle fatigue using the M-waves elicited by the additional pulse. Values of FI_*S*_(*n*) are shown for the cycles after the 30th cycle because of muscle force potentiation.

Figure 13 shows calculated values of FI_*M*_(*m*) comparing with values of FI_*S*_(*n*) for each subject. The results show that FI_*M*_(*m*) provided good estimation of FI_*S*_(*n*). As shown in Table 1, absolute values of difference between FI_*S*_(*n*) and FI_*M*_(*m*) were less than 0.08. Values of correlation coefficient between those indexes were larger than 0.94 for 2 subjects who showed large amount of muscle fatigue, while they were larger than 0.69 for 2 subjects who showed a little fatigue.

## 5. Discussions

This study demonstrated that M-waves elicited by the 2nd pulses of double pulses would be effective in muscle fatigue evaluation for repetitive movements in rehabilitation training using FES, while the M-wave obtained from single pulse was not sensitive to detect muscle fatigue. Considering M-waves elicited by the 2nd pulse of a double pulse, its peak-to-peak amplitude decreased in early cycles as muscle force decreased under the isometric condition, which showed strong correlation between conventional and proposed fatigue indexes. Furthermore, fatigue index FI_*M*_(*m*) showed good estimation of conventional-type index FI_*S*_(*n*) during repetitive movement control. These suggest that the proposed fatigue index using M-wave can be used as one of the alternatives to the conventional index in order to evaluate muscle fatigue in repetitive movement training with FES. Since the conventional fatigue index generally needs force measurement with a specialized measurement device (e.g., isometric torque measurement), it is hard to evaluate muscle fatigue during movement, especially in ambulatory application of FES such as walking. Monitoring the fatigue development by assessing M-waves would be a practical solution convenient for such FES applications.

**Figure 14:**
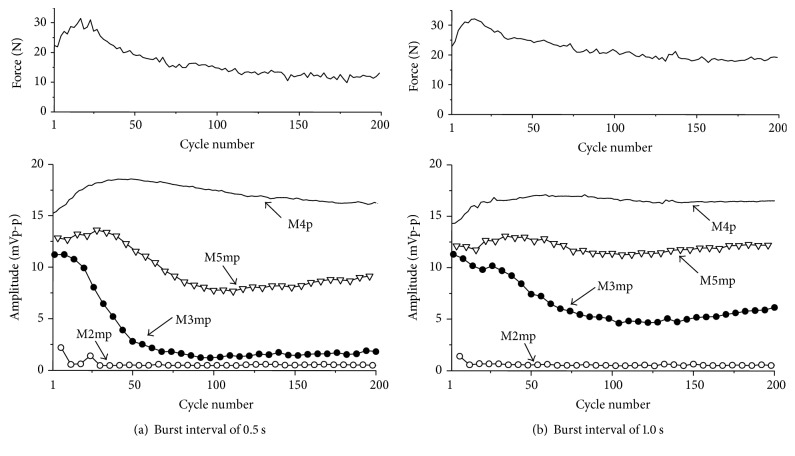
A result of preliminary test with different time intervals between stimulation bursts.

Amplitude of M-wave elicited by a single pulse in a stimulation burst was not sensitive to detect muscle fatigue during repeated activation by FES. It has been found that the decreasing of the amplitude of the M-waves elicited by the single pulse during intermittent electrical stimulations was much smaller than the decreasing during the sustained electrical stimulation [19]. The result of this paper that suggested unusefulness of FI_4_(*m*) in repetitive FES movements is similar to the points suggested in previous studies [13, 19]. On the other hand, M-wave amplitude elicited by a single pulse was effective to evaluate muscle fatigue for long lasting continuous constant electrical stimulation under the isometric condition [11].  Figure 14 shows an example of results of preliminary test with different time intervals between stimulation bursts. Amplitude of M-wave elicited by a single pulse decreased as the number of stimulation bursts increased in the case of stimulation burst interval of 0.5 s, while the decrease of the M-wave amplitude was small with the time interval of 1 s. Since the membrane potential of human muscle fibers recovered over 1 s [20], the time interval of 1 s was suggested to recover action potential production. M-wave elicited by a single pulse may be useful in more severe fatigue condition.

M-waves elicited by the 2nd pulse of a double pulse showed clear decrease during repetitive stimulation conditions. Double pulse stimulation is sometimes used to measure the refractory period, and large size of motor units is known to have shorter refractory period than small size of motor units [21, 22]. In addition, generally, large size of motor unit innervates faster muscle fibers that are more fatigable than slow muscle fibers that are innervated by small size of motor units [23]. Therefore, it is considered that M-waves elicited by the 2nd pulse with shorter IPI are produced mainly by large size of fatigable motor units. This suggests that M-waves elicited by the 2nd pulse with shorter IPI decrease rapidly in earlier cycles than those with longer IPIs because of earlier muscle fatigue.

**Figure 15:**
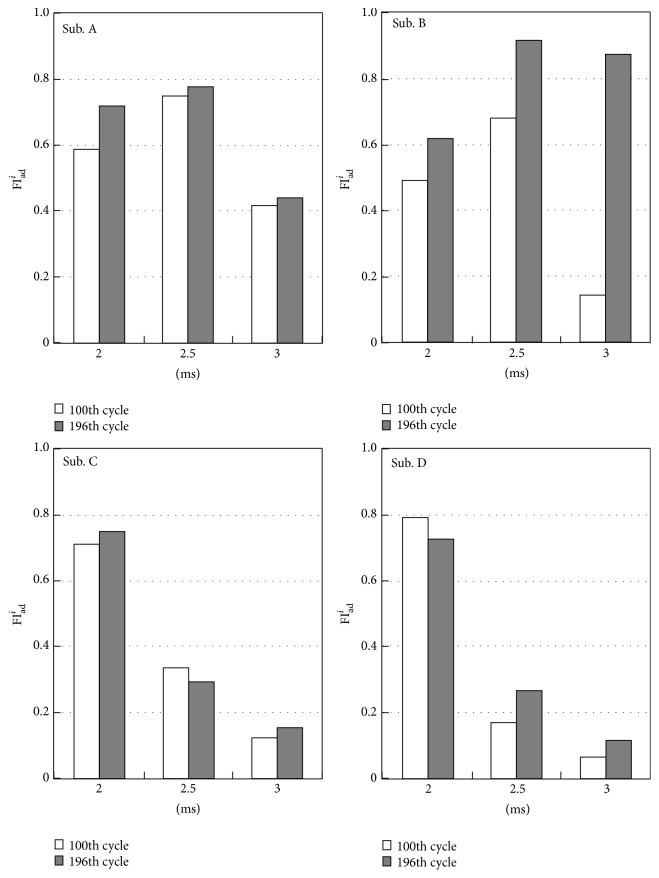
Comparison results of FI_ad_^*i*^ at the 100th cycle and at the 196th cycle in the knee angle control.

Fatigue indexes FI_ad_^2^, FI_ad_^2.5^, and FI_ad_^3^ obtained from M-waves elicited by double pulses with different IPIs were shown to have a possibility of evaluating muscle fatigue resistance in more detail. As seen in Figures 12 and 13, subjects A and B showed larger values of fatigue indexes than those of subjects C and D. These differences in fatigue indexes are considered to show the difference in fatigue resistance between subjects.  Figure 15 shows values of FI_ad_^*i*^ at the 100th and the 196th cycles for each subject in the knee angle control. Subjects A and B showed large values of all the indexes, while subjects C and D showed large value only for FI_ad_^2^. It is found that increase of fatigue index FI_*M*_(*m*) shown in subjects A and B was caused mainly by increase of FI_ad_^2.5^ and FI_ad_^3^. The increase in FI_ad_^*i*^ is considered to relate to the decrease of the number of activated motor units that have shorter refractory period than its IPI. Large values of FI_ad_^*i*^ of subjects A and B suggest that most of motor units in the muscle fatigued in the knee extension angle control, while the fatigable motor units with shorter refractory period mainly fatigued in case of subjects C and D.

FI_*M*_ was shown to be an effective fatigue index in intermittent repetitive movement training by FES. It is inappropriate to use FI_*S*_ because the muscle sensitivity is affected by movements of other parts of the body or the change of posture. The experimental condition of this paper excluded those problems in the sensitivity by testing muscle fatigue evaluation in simple exercise of knee extension at the sitting position. For muscle fatigue evaluation in various movement conditions, fatigue indexes obtained from directly measured muscle activity (M-waves) would be effective. Particularly, M-waves elicited by double pulses are expected to provide useful information in repetitive movement training by FES for motor rehabilitation.

Paralyzed muscles become smaller and weak generally because the muscle fibers transform into faster glycolytic types, and they are therefore highly fatigable [24–27]. Electrical stimulation training incorporating resistance training can increase the size and strength of paralyzed muscles [25, 26, 28]. A transformation of fiber types after electrical stimulation training has also been reported [25, 28]. In order to evaluate these changes, the methods such as the collection of muscle biopsies or the prolonged measurement of EMG signals under the isometric condition are required. The measurement of the M-waves elicited by double pulses has a possibility of evaluating these changes easily and is expected to help to evaluate the therapeutic effect in motor rehabilitation with FES.

The results of this paper were obtained from 4 neurologically intact subjects. As shown in Figure 14, decrease of M-wave amplitude during fatiguing under the repetitive stimulation condition also depends on stimulation burst interval. In this paper, the time interval between stimulation bursts was determined as 1 s because 0.5 s interval was considered to be short for the interval of movement training in rehabilitation and muscle fatigue occurred with the interval of 1 s. Since the M-waves elicited by the double pulses were found to be effective for muscle fatigue evaluation under repetitive stimulation conditions, it would be desired to perform more tests with different burst intervals and various IPIs increasing the number of subjects.

## 6. Conclusion

M-waves elicited by double pulses were measured under the conditions of repeated intermittent stimulation for training with FES, which were force production under the isometric condition and joint angle control based on cycle-to-cycle control under the dynamic movement condition. The amplitude of the M-wave elicited by the additional pulse constituting a double pulse showed decrease in the early cycles depending on the IPI of double pulse as the muscle fatigue increased. The M-wave amplitude elicited by the single pulse in a burst was not sensitive to detect muscle fatigue caused by repeated activation by FES with stimulation interval of 1 s. Fatigue index using the M-waves elicited by the 2nd pulses of double pulses was demonstrated to estimate muscle fatigue appropriately during movements by repetitive burst electrical stimulation. It is expected that M-waves elicited by the 2nd pulses provide the effective information of muscle fatigue during repetitive activation by FES in rehabilitation training.

## Figures and Tables

**Table 1:** Absolute values of difference and values of correlation coefficient between FI_*S*_(*n*) and FI_*M*_(*m*).

Subject	Absolute difference	Correlation coefficient
A	0.060 ± 0.047	0.947
B	0.024 ± 0.017	0.990
C	0.072 ± 0.066	0.814
D	0.072 ± 0.047	0.688

## References

[1] Glanz M., Klawansky S., Stason W., Berkey C., Chalmers T. C. (1996). Functional electrostimulation in poststroke rehabilitation: a meta-analysis of the randomized controlled trials. *Archives of Physical Medicine and Rehabilitation*.

[2] Yan T., Hui-Chan C. W. Y., Li L. S. W. (2005). Functional electrical stimulation improves motor recovery of the lower extremity and walking ability of subjects with first acute stroke: a randomized placebo-controlled trial. *Stroke*.

[3] Sheffler L. R., Chae J. (2007). Neuromuscular electrical stimulation in neurorehabilitation. *Muscle and Nerve*.

[4] Thrasher T. A., Zivanovic V., McIlroy W., Popovic M. R. (2008). Rehabilitation of reaching and grasping function in severe hemiplegic patients using functional electrical stimulation therapy. *Neurorehabilitation and Neural Repair*.

[5] Weingarden H., Ring H. (2006). Functional electrical stimulation-induced neural changes and recovery after stroke. *Europa Medicophysica*.

[6] Bogataj U., Gros N., Kljajic M., Acimovic R., Malezic M. (1995). The rehabilitation of gait in patients with hemiplegia: A comparison between conventional therapy and multichannel functional electrical stimulation therapy. *Physical Therapy*.

[7] Merletti R., Knaflitz M., De Luca C. J. (1992). Electrically evoked myoelectric signals. *CRC Critical Reviews in Biomedical Engineering*.

[8] Mizrahi J., Levy M., Ring H., Isakov E., Liberson A. (1994). EMG as an indicator of fatigue in isometrically FES-activated paralyzed muscles. *IEEE Transactions on Rehabilitation Engineering*.

[9] Tepavac D., Schwirtlich L. (1997). Detection and prediction of FES-induced fatigue. *Journal of Electromyography and Kinesiology*.

[10] Yu N. Y., Chen J.-J. J., Ju M. (1999). Study of the electrically evoked EMG and torque output during the muscle fatigue process in FES-induced static and dynamic contractions. *Basic and Applied Myology*.

[11] Watanabe T., Miura N., Hoshimiya N., Handa Y. (2000). The possibility of using M-waves related to double pulses for evaluating muscle fatigue in FES control. *Transactions of Japanese Society for Medical and Biological Engineering*.

[12] Kojović J., Djurić-Jovičić M., Došen S., Popović M. B., Popović D. B. (2009). Sensor-driven four-channel stimulation of paretic leg: functional electrical walking therapy. *Journal of Neuroscience Methods*.

[13] Estigoni E. H., Fornusek C., Smith R. M., Davis G. M. (2011). Evoked EMG and muscle fatigue during isokinetic FES-cycling in individuals with SCI. *Neuromodulation*.

[14] Chen J.-J. J., Yu N.-Y. (1997). The validity of stimulus-evoked EMG for studying muscle fatigue characteristics of paraplegic subjects during dynamic cycling movement. *IEEE Transactions on Rehabilitation Engineering*.

[15] Miura N., Watanabe T., Sugimoto S., Seki K., Kanai H. (2011). Fuzzy FES controller using cycle-to-cycle control for repetitive movement training in motor rehabilitation. Experimental tests with wireless system. *Journal of Medical Engineering and Technology*.

[16] Miura N., Watanabe T., Kanai H. A preliminary test of muscle fatigue evaluation using M-wave for rehabilitation with electrical stimulation.

[17] Bajd T., Kralj A., Turk R., Benko H. (1990). Symmetry of FES responses in the lower extremities of paraplegic patients. *Journal of Biomedical Engineering*.

[18] Eom G.-M., Watanabe T., Hoshimiya N., Khang G. (2002). Gradual potentiation of isometric muscle force during constant electrical stimulation. *Medical and Biological Engineering and Computing*.

[19] Hainaut K., Duchateau J. (1989). Muscle fatigue, effects of training and disuse. *Muscle and Nerve*.

[20] Z'Graggen W. J., Bostock H. (2009). Velocity recovery cycles of human muscle action potentials and their sensitivity to ischemia. *Muscle and Nerve*.

[21] Horcholle-Bossavit G., Jami L., Petit J., Scott J. J. A. (1987). Activation of cat motor units by paired stimuli at short intervals. *Journal of Physiology*.

[22] Villagran-Vargas E., Rodríguez-Sosa L., Hustert R., Blicher A., Laub K., Heimburg T. (2013). Variations in interpulse interval of double action potentials during propagation in single neurons. *Synapse*.

[23] Burke R. E., Levine D. N., Tsairis P., Zajac F. E. (1973). Physiological types and histochemical profiles in motor units of the cat gastrocnemius. *Journal of Physiology*.

[24] Grimby G., Broberg C., Krotkiewska I., Krotkiewski M. (1976). Muscle fiber composition in patients with traumatic cord lesion. *Scandinavian Journal of Rehabilitation Medicine*.

[25] Rochester L., Chandler C. S., Johnson M. A., Sutton R. A., Miller S. (1995). Influence of electrical stimulation of the tibialis anterior muscle in paraplegic subjects. 1. Contractile properties. *Paraplegia*.

[26] Rochester L., Barron M. J., Chandler C. S., Sutton R. A., Miller S., Johnson M. A. (1995). Influence of electrical stimulation of the tibialis anterior muscle in paraplegic subjects. 2. Morphological and histochemical properties. *Paraplegia*.

[27] Gerrits H. L., De Haan A., Hopman M. T. E., Van Der Woude L. H. V., Jones D. A., Sargeant A. J. (1999). Contractile properties of the quadriceps muscle in individuals with spinal cord injury. *Muscle and Nerve*.

[28] Crameri R. M., Weston A., Climstein M., Davis G. M., Sutton J. R. (2002). Effects of electrical stimulation-induced leg training on skeletal muscle adaptability in spinal cord injury. *Scandinavian Journal of Medicine and Science in Sports*.

